# Wireless Sensor Network Security Enhancement Using Directional Antennas: State of the Art and Research Challenges

**DOI:** 10.3390/s16040488

**Published:** 2016-04-07

**Authors:** Daniel-Ioan Curiac

**Affiliations:** Automation and Applied Informatics Department, Politehnica University of Timisoara, V. Parvan No. 2, Timisoara 300223, Romania; daniel.curiac@aut.upt.ro; Tel.: +40-256-403-227; Fax: +40-256-403-214

**Keywords:** wireless sensor networks, directional antenna, security risks, malicious attacks

## Abstract

Being often deployed in remote or hostile environments, wireless sensor networks are vulnerable to various types of security attacks. A possible solution to reduce the security risks is to use directional antennas instead of omnidirectional ones or in conjunction with them. Due to their increased complexity, higher costs and larger sizes, directional antennas are not traditionally used in wireless sensor networks, but recent technology trends may support this method. This paper surveys existing state of the art approaches in the field, offering a broad perspective of the future use of directional antennas in mitigating security risks, together with new challenges and open research issues.

## 1. Introduction

Wireless sensor networks (WSNs) have emerged as a key technology for a broad spectrum of applications, ranging from weather forecasting [1] or complex industrial plant monitoring [2] to military surveillance [3]. These types of cyber-physical systems are prone to various malicious attacks which theoretically originate from three different causes: (i) the limited power, communication and computational resources of the nodes; (ii) the unattended and hostile environments where they are often deployed; and (iii) the open nature of the wireless transmission medium. In order to cope with security related issues, besides already traditional approaches like message encryption or node authentication, a convenient solution arises: equipping the sensor nodes with directional antennas.

Usually, sensor nodes employ omnidirectional antennas for wireless communication due to a variety of reasons including their small size, low cost, ease of deployment, simplified transmission-related protocols, *etc*. With the advancements of smart antenna technology, the omnidirectional antennas may either be replaced by directional ones or can work in tandem with them on the same motes. The advantages brought by directional antennas to WSN nodes can be seen not only in increased quality of transmissions, optimization of energy usage, decreased number of hops due to longer transmission range, but also from the security point of view.

Directional antennas can mitigate the malicious attack risks in WSNs in two ways: (a) directly, by being immune to attacks launched from outside their narrow radiation region; or (b) indirectly based on node position verification—here a node equipped with directional antenna, using the received signal’s direction of arrival to compute the position of a sender node (in conjunction with other trusted nodes or beacons), can identify malicious nodes by checking their position against a trusted list. By using these two lines of defense against hostile attacks, the nodes equipped with directional antennas may identify, mitigate or even eliminate security risks when speaking about eavesdropping, jamming, wormhole attacks or Sybil attacks. From this perspective, this paper aims to survey the current state of the art in the field and to identify the major research challenges and perspectives.

The remainder of the paper is organized as follows: Section 2 presents a brief comparison of the two antenna types—omnidirectional and directional, while Section 3 is devoted to small directional antenna prototypes that can equip sensor nodes. The state of the art in coping with security attacks using directional antennas is surveyed in Section 4. Section 5 reveals the research challenges and opportunities of employing directional antennas to reduce security risks, while conclusions are drawn in Section 6.

## 2. Directional and Omnidirectional Antennas—A Brief Comparison

**Figure 1:**
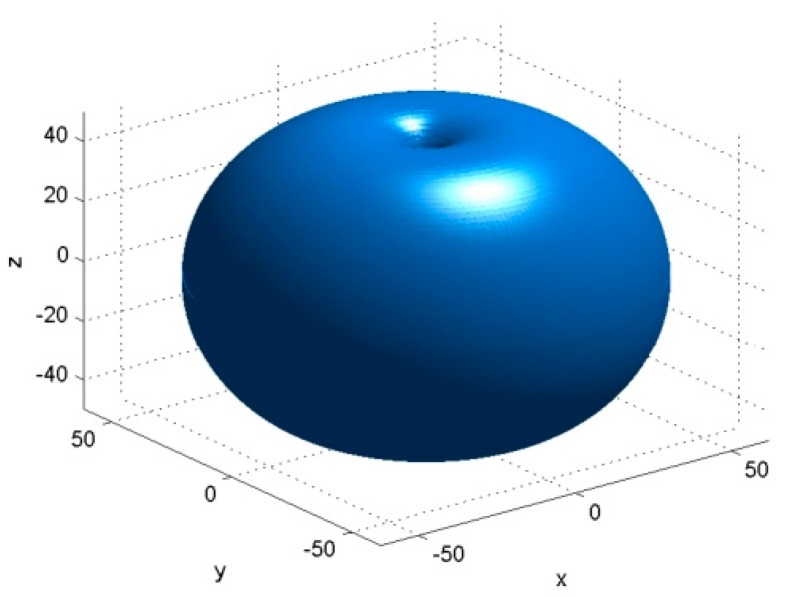
Omnidirectional radiation pattern (dipole antenna).

Traditionally, communication inside WSNs is done using omnidirectional antennas which broadcast radio signal almost uniformly in all directions. Omnidirectional antennas are small, inexpensive and simply to deploy, but they suffer from poor spatial reuse, high collisions, reduced energy efficiency and are susceptible to security attacks [4,5]. A relevant example of omnidirectional antennas is a simple dipole, having the radiation pattern depicted in Figure 1.

**Figure 2:**
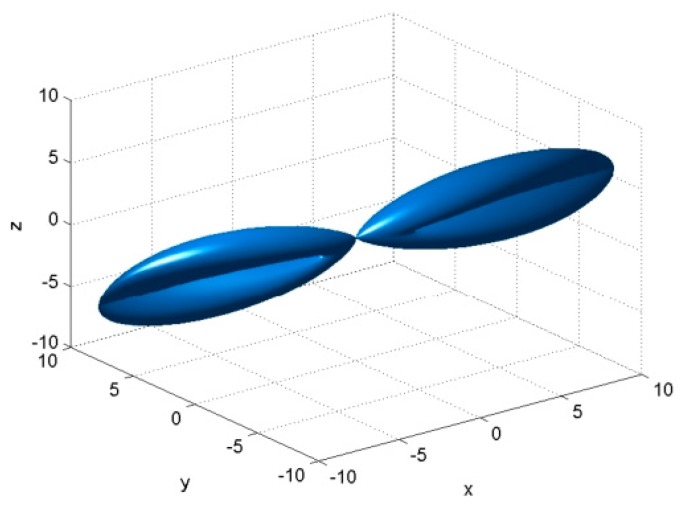
Directional radiation pattern for the binomial array antenna.

If the mentioned drawbacks dramatically affect the normal WSN operation and security, wireless nodes can be equipped with directional antennas either alone or in conjunction with existing omnidirectional antennas [6]. A directional antenna, also known as beam antenna, is the type of antenna which emits or receives greater power in a particular direction (Figure 2) [7,8]. By focusing its radiation pattern in a specific direction, they reduce the interferences and collisions, increase the gain and enhance the security against eavesdropping, jamming or other malicious attacks.

A brief comparison between the two types of antennas [9] is provided in Table 1, highlighting three aspects of practical interest for directional antennas usage in future WSN technologies:

- Improved energy consumption; the wireless data transmission is proved to be the most energy-intensive operation of a sensor node [10,11]. By focusing their transmitted power in the needed direction, directional antennas have the potential to reduce the energy usage [12] and therefore to extend sensor nodes’ lifetime.
- Longer transmission range; reporting information inside WSNs using fewer hops [13,14] or reducing the risk for nodes or groups of nodes to become isolated (due to malfunctions, battery depletion or malicious attacks) [15] can significantly improve the WSN performance, when using directional antennas.
- Higher security; derived either from their immunity to eavesdroppers [16] or jammers [17] placed outside their narrow radiation region or from the feature of determining the exact position of a sender node using the signal’s angle of arrival [18], the directional antennas can mitigate the risk of security attacks.

Directional antennas are generally constructed by combining simple antenna elements (e.g., dipoles) into antenna arrays. Their overall radiation patterns are influenced by the type, the number and the geometrical configuration of the elements and also by the characteristics of the signal applied to each element. There are basically two types of directional antennas: traditional directed antennas and smart antennas. Traditional directed antennas [19] (e.g., Yagi-Uda, helix, aperture horn, reflector, patch antennas, *etc*.) have a fixed beam that can be oriented in the desired direction by mechanical rotation. Smart antenna [20] is a generic name that describes an antenna array endowed with digital signal processing techniques, which automatically optimize its radiation/reception pattern. Smart antennas can be classified [21,22] as either switched beam or adaptive array systems. A switched beam antenna [23] can generate multiple fixed beams, automatically switching from one beam to another every time when needed. The second type of smart antennas—adaptive array systems [24]—possess the ability to actively locate and track desired signal in order to dynamically mitigate interferences, optimizing the signal reception.

## 3. Directional Antennas Suitable for WSN Nodes

The physical layer of a wireless sensor network is in charge of bit-stream transmission/reception over wireless communication channels, performing a series of tasks that includes carrier frequency selection and generation, signal detection, modulation or data encryption. A central role in this context is played by antenna devices which basically transform electric power into electromagnetic waves, or *vice versa*.

In order to be used in WSN nodes, directional antennas have to possess four basic features: they must be small, reasonably priced, consume low power and able to operate in licensed frequency bands: 315 MHz, 433 MHz or 868 MHz in Europe, 915 MHz in North America, 2.45 GHz Industrial-Scientific-Medical (ISM) band or within the millimeter-wave spectrum [25,26,27,28]. These requirements drastically limit the number of directional antenna construction types adaptable for sensor nodes [29].

The directional antenna prototypes specifically designed to equip sensor nodes are briefly presented in Table 2.

Leang and Kalis [30] indicated the need and usefulness of smart antenna integration into WSN nodes by analyzing the overall network performance and nodes’ power consumption. They proposed a small, inexpensive and modular sensor node hardware platform, termed SensorDVB. This platform, built from commercial-off-the-shelf components and occupying no more than 33 cc in volume, provided onboard processing, sensing, and radio communication using smart antennas operating in the 868 MHz radio frequency spectrum.

Nilsson [31,32] identified three construction types as plausible candidates to equip WSN nodes: the adcock-pair antenna, pseudo-Doppler antenna, and electronically switched parasitic element antenna. He proposed a variant of electronically switched parasitic element antenna, named SPIDA 2.44-GHz prototype, and demonstrated its efficiency through numerical simulations and lab experiments. Öström *et al.* [38] presented a real-world evaluation of the SPIDA prototype. They interfaced this electronically switched directional antenna with a TMote Sky (an off-the-shelf sensor node), obtaining a fully functional real-world WSN node with improved performances in terms of communication range, and wireless link quality.

A 2.4 GHz four-beam patch antenna prototype meeting the size, cost and energy constraints of sensor nodes was proposed by Giorgetti *et al.* [33]. This directional antenna uses a box-like structure of four coaxially fed planar patch antennas. Experiments involving TelosB motes demonstrated the substantial benefits of using such antennas, the communication range being extended from 140 to more than 350 m while suppressing the interferences due to multipath fading.

Liang *et al.* [34] developed a beam-switching WSN node using the VirtualSense platform. They enclosed the VirtualSense mote with an active cylindrical frequency selective surface. By this action, the antenna’s radiation pattern was converted from omnidirectional into a directional one by modifying the configuration of active PIN diodes. As a direct result, the miniaturization and ultra-low-power features of the VirtualSense node were preserved.

Catarinucci *et al.* proposed and tested two cost-effective and compact switched-beam antenna prototypes, in the ISM band. The first one employs a radiation structure made of eight microstrip antennas using rectangular two-element patch antenna arrays and a vertical half-wavelength dipole antenna [12]. The second prototype [35,36] uses a group of four identical antennas containing two quarter-wavelength l-shaped slot antenna elements which are disposed in a symmetrical planar structure of 10 × 10 cm^2^.

Another directional antenna prototype for WSN nodes was developed and evaluated in a fully directional neighbor discovery protocol by Felemban *et al.* [37]. For this, they equipped five sensor nodes, developed on a Nano-Qplus hardware platform, with low-cost 6-sectored antennas having an overlap of 120 degrees in azimuth.

Although the research in developing directional antennas suitable for WSN nodes is in the early phases, the results obtained so far are encouraging. This allows us to envisage new models of wireless communications between sensor nodes, endowed with higher security.

## 4. Security Benefits of Directional Antennas in WSNs

Directional antennas can mitigate or even eliminate the risks related to some categories of security attacks on WSNs due to their specific radiation pattern which can be materialized into mechanisms for localization of neighboring or malicious nodes, or can drastically reduce the areas from where an attack can be carried out. The main types of attacks that can be mitigated using directional antennas are: eavesdropping, jamming, Sybil attack and wormhole attack, but similar countermeasures can reduce the risks for traffic analysis, man-in-the-middle attack or node capturing attack.

Directional antennas can reduce malicious attack risks in two ways, either directly by being immune to attacks launched from outside their narrow radiation region, or indirectly based on position verification procedures [39,40] employing the received signal’s direction of arrival. The main research in the field is briefly presented in Table 3 and discussed in the following subsections.

### 4.1. Eavesdropping

**Figure 3:**
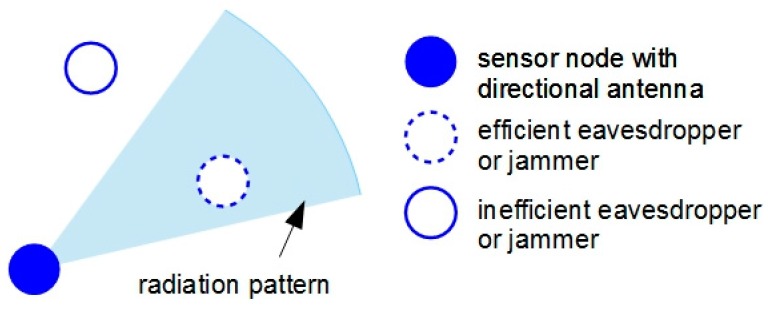
Eavesdropping and jamming effect with directional antennas.

Eavesdropping is the attack in which a malicious entity intercepts private communication in an unauthorized real-time manner. The attacker, analyzing the stolen information packets can obtain contextual and targeted information (e.g., sensing data, network routing paths, *etc.*) that later can be used in more destructive attacks. In WSNs, two categories of eavesdropping attacks have been identified [55,56]: (a) passive eavesdropping where malicious nodes intercept the information by simply listening to the wireless broadcast messages; and (b) active eavesdropping in which malicious nodes pretending to be friendly nodes gather the information by sending queries to the network nodes or access points. In the case of sensor nodes equipped with directional antennas, efficient eavesdroppers are those placed inside the antennas’ radiation regions (Figure 3).

In [41,42], Dai *et al.* proved that when using directional antennas, the eavesdropping probability is drastically decreased compared to the case of omnidirectional antennas. These studies make three important contributions: (i) they establish eavesdropping models for both omnidirectional and directional antennas in the context of wireless sensor networks; (ii) they prove that in the case of directional antennas the eavesdropping probability is diminished due to two factors: the number of hops to route a message is reduced and the exposure region from where malicious nodes may listen is smaller; and (iii) they validate the two eavesdropping models (in omnidirectional and directional case) and the corresponding values of eavesdropping probability through extensive simulation studies.

Another analysis of the effects of using directional antennas upon eavesdropping probability in wireless networks, but this time from the attacker’s perspective, is presented by Li *et al.* [43]. The proposed framework enables the theoretical evaluation of the node density and antenna model on eavesdropping possibility, furthermore laying the foundation for cost-effective and practical eavesdrop attacks prevention mechanisms.

An interesting approach to mitigate eavesdropping attacks in wireless networks is proposed in [44] and employs defensive jammers. These devices are meant to confine the network’s wireless coverage into a spatially limited zone by increasing the interference level outside that particular area. By this, a potential adversary located outside the coverage zone will be blocked from illegitimately gathering the sent messages. The results of this defense strategy are substantially improved, even in the case of advanced attackers that use anti-jamming countermeasures, if these defensive jammers are placed in optimal locations and use directional antennas.

### 4.2. Jamming

Jamming is the deliberate act of broadcasting an inference radio signal aimed to disrupt wireless communication. This type of electromagnetic interference can be accomplished either in a simple manner when the jammer continually transmits interference signals or using more sophisticated approaches based on communication protocol vulnerabilities.

Due to their particular radiation patterns, directional antennas can efficiently mitigate the effects of jamming attacks being able to safely communicate if the jammer’s location is outside antenna’s coverage sector (Figure 3). The scientific literature reveals some significant works in this domain.

In [45], Noubir studied the effects of jamming attacks in a multihop *ad hoc* communication network. By comparing the network connectivity index when either omnidirectional or directional antennas are used in jamming circumstances, the author proved a significant improvement in the second case. This result stands not only for randomly placed jammers but also for jammers optimally located in the network area. Moreover, the result can be extrapolated to diverse types of smart antennas able to concentrate the beam’s power in the receiver’s direction (e.g., sectored antenna or beamforming antenna) [57,58].

For mitigating the jamming effect in wireless sensor networks, Panyim *et al.* [46] proposed a combined strategy that uses pre-distributed cryptographic keys in conjunction with sensor nodes able to switch from omnidirectional to directional antennas anytime a jamming attack is detected.

In order to reduce unwanted interferences in randomly deployed wireless sensor networks Staniec and Debita [47] suggested two possible solutions: equipping the nodes with directional antennas and establishing a superior limit of the duty cycle for each network node. While the first solution decreases the spatial area from where a jamming attack can be launched, the other decreases the temporal interval when a malicious attack can affect the node.

### 4.3. Sybil Attack

Usually, in a wireless sensor network each node has its own identity (ID), a one-to-one relationship between nodes and their unique IDs being a prerequisite for many network mechanisms [59]. In a Sybil attack [60], a malicious node forges the identities of authenticated network nodes and, as a consequence, can spread its aggressive activities to other nodes or even throughout the entire network.

**Figure 4:**
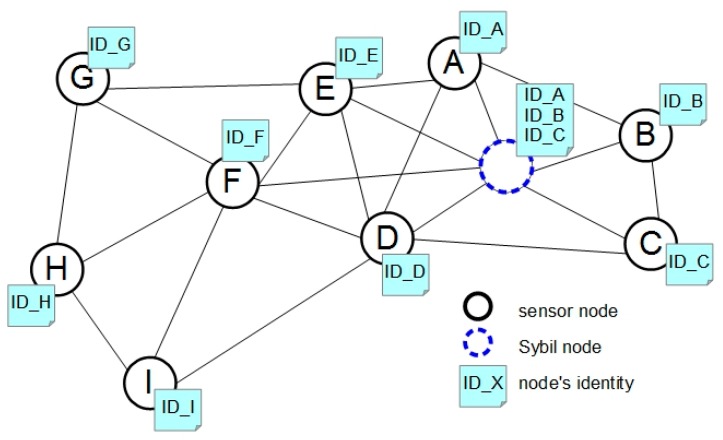
Sybil attack in WSN.

An example of such an attack is presented in Figure 4, where the Sybil node, shown in dotted line, uses the identity of three network nodes (A, B and C) to maliciously alter the nodes’ normal behavior.

Newsome *et al.* [48] identified the ways Sybil attacks can be used to disrupt WSN operations implying distributed storage, routing algorithms, data aggregation mechanisms, voting algorithms, fair resource allocation and misbehavior detection. They provide a list of possible defenses which include node validation and authentication, resource testing (computation, storage, or communication resource testing), random key predistribution, identity registration and position verification.

From this comprehensive list, one of the most efficient methods to discover the Sybil nodes is undeniably the position verification technique. Accordingly, the Sybil nodes can be identified by comparing their exact position with the previously known locations of network nodes from which the Sybil nodes stole the identities. This type of methods usually employs two elements: (a) radio signal characteristics (signal strength and/or direction); and (b) trusted nodes cooperation for node identification and authentication. Directional antennas are inherently offering the direction of captured signals. If two messages coming from two nodes having the same IDs are concurrently gathered from two different directions, then we can come to a logical decision: one of the two network nodes is undoubtedly malicious. Such a methodology can be derived from the one described in [49], which uses nodes equipped with GPS devices and directional antennas. Thus, the precise location of all WSNs components are known, while the position of Sybil nodes may be calculated using triangulation [61] based on information captured by directional antennas and by employing the cooperation of at least one trusted node.

A simplified Sybil attack named evil-twin, in which the malicious node is using only one stolen identity, is addressed by Bhatia *et al.* [50] using four-sector directional antennas. If two messages with the same sender ID come from two different angles a logical conclusion is drawn: one of them is bogus. Subsequently an algorithm named Hyperbolic Position Bounding (HPB) [62] is employed to obtain the location of the two twin nodes (the real node and the malicious node).

Approaches for coping with Sybil attacks in wireless sensor networks based on directional antennas can also be derived from methods proposed for other types of wireless networks. For example, some methods developed for mobile *ad hoc* networks (MANETs) or one of their subcategories (vehicle *ad hoc* networks—VANETs) can be simply particularized to address the Sybil attack in WSNs. Two such methods attracted our interest.

Vaman and Shakhakarmi [51] proposed an integrated key (a type of cryptographic key that encloses a symmetric node’s ID, geographic location of the node and round trip response time)-based Strict Friendliness Verification (SFV) of neighboring nodes. As a result, a set of verifier nodes discover the Sybil nodes by dynamically changing the symmetric node’s ID every time a new wireless connection is established, and by encrypting/decrypting each packet by different integrated keys.

A cross-layer scheme to detect Sybil attacks in VANETs is proposed in [52]. A trial packet is sent to the mobile node’s claimed location employing a directional antenna. If the mobile node is in the claimed position, it can receive the packet and reply with a response packet. The identification of Sybil attack is based on directional information of the exchanged messages, coupled with the public key cryptography and hash function applied to the same messages.

### 4.4. Wormhole Attack

**Figure 5:**
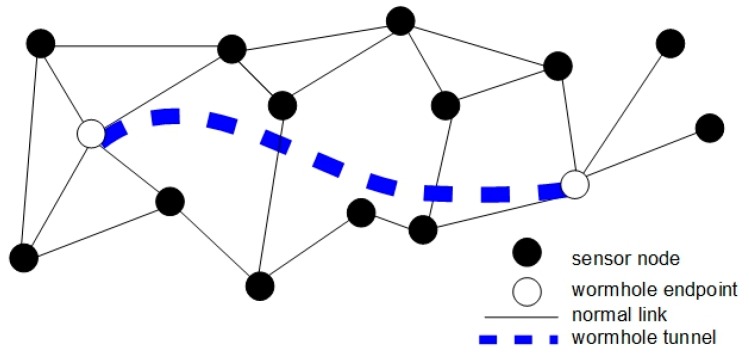
Wormhole attack.

The wormhole attack [63,64] occurs on the network or physical layer and is classified as severe due to the fact that no cryptographic information is needed. This attack involves two malicious nodes that establish a uni- or bi-directional low latency link among them in order to shortcut the regular transmission path (Figure 5). By this, the adversary can collect, analyze, drop and modify the packets or can change the network topology by creating the illusion that the two ends of the wormhole tunnel are very close to each other.

The methods developed to identify the wormhole attacks usually require that all/some nodes be equipped with extra hardware [65]. When the radio transmissions inside a WSN are done using directional antennas, the wormhole attack can be discovered based on direction of the received signals that will help the nodes maintain an accurate list of their neighbors.

The three approaches with different levels of wormhole attack mitigation, proposed by Hu and Evans [53], assume that all the network nodes are equipped with directional antennas. The basic idea is to maintain an accurate list of neighbors for each network node and on this basis to reject the communication links that lead to wormhole end-points. This way, the wormhole transmitters are recognized as fake neighbors and the network will ignore them. The two authors assume an antenna model with N zones, where each zone is characterized by a conical radiation pattern covering an angle of 2π/N radians. When idle, the sensor node works in omnidirectional antenna mode until a message is received. By determining the zone with the maximal signal power, the node is able to switch to a directional antenna mode for communicating with message’s sender. The three increasingly effective protocols presented by Hu and Evans [53], are:

(a) The directional neighbor discovery protocol. The proposed mechanism does not rely on any type of cooperation among nodes. The protocol works in three consecutive steps: (i) a node (called announcer) of a just-deployed sensor network sends a HELLO-type message including its ID; (ii) all nodes that receive the HELLO message reply with an encrypted message that basically contains their node ID and the zone where the message was received. The encryption process is done using previously established keys, stored on each node together with corresponding neighbor ID; and (iii) the announcer will decrypt the message verifying the node ID and that the zone reported by the neighbor is opposite to its zone. After the neighbor discovery process is finished, the node will ignore any kind of messages coming from nodes that do not belong to the neighbor list. Even its effect on mitigating the wormhole attacks is reduced, the protocol is envisioned by the two authors to represent a strong basis for the following two.
(b) The verified neighbor discovery protocol is based on sharing information between network nodes. It can stop attacks in which the malicious entity controls the two wormhole endpoints and when the targeted nodes have no direct communication link (are at least two hops distant). The mechanism is based on directional neighbor discovery protocol which is enhanced by a verification procedure done using verifiers (network nodes that are not in opposite direction from the wormhole endpoints). The role of verifier-nodes is to check the legitimacy of announcers.
(c) The strict neighbor discovery protocol adds a supplementary requirement (the verifier region must be empty when two nodes are out of radio range) for verifier-nodes to cope with Worawannotai attack (the malicious entity convinces two close and non-neighboring nodes that they are neighbors [53]), too.

This ensemble of three protocols can countermeasure the wormhole attacks without clock synchronization among nodes or precise location information. Shi *et al.* [54] proposed a Secure Neighbor Discovery (SND) scheme for wireless networks with a centralized network controller (NC). The scheme consists of three stages: NC broadcasting phase; network node response/authentication; and, NC time-delay analysis. By using signature based authentication, transmission time information and antenna direction information, the SND scheme can efficiently prevent and detect the wormhole attacks.

Another method that uses directional information gathered by trusted nodes to cope with wormhole attack is described in the case of MANETs [51], but can be also used in WSNs. The mechanism in based on symmetric node IDs, round trip response times and real time location information obtained by directional antennas. These data, encapsulated in integrated cryptographic keys are used in a Strict Friendliness Verification (SFV) of neighbors protocol, before multi hop packet routing.

## 5. Challenges and Perspectives

Although the use of sensor nodes equipped with directional antennas represents a promising tool in mitigating the risks of security attacks in WSNs, research in this field is still in the beginning stages. This research status should be significantly improved with the availability of new commercial-off-the-shelf sensor nodes equipped with directional antennas and relying on efficient network protocols.

However, the road towards endowing commercial wireless sensor nodes with directional antennas is still long and not free of challenges, and further improvements being expected in both technological and operational aspects. The most important difficulties in providing such sensor nodes lie in:

(i) designing small sized, reasonably priced and energetic-efficient directional antennas able to be integrated in highly resource-constrained sensor nodes;
(ii) developing efficient MAC protocols to address deafness, directional Hidden Terminal (HT) problem or Head-of-Line (HoL) blocking problem in multi-hop wireless networks [66,67];
(iii) providing network protocols able to assure self-localization, self-configuration, self-synchronization and self-optimization in the case of randomly deployed sensor networks using aerial scattering or other similar procedures;
(iv) designing effective and reliable neighbor discovery mechanisms, being known that traditional approaches either depend on omnidirectional announcers and on time synchronization or are two complex to be implemented in real large-scale sensor networks [13,37];
(v) adapting the in-network data and message aggregation mechanisms to the directional antenna-based topology of WSN;
(vi) designing customized topology control mechanisms to increase effective network capacity and conserve energy; and
(vii) providing appropriate QoS models incorporating both communication-related parameters (e.g., delay, packet delivery ratio, jitter, *etc*.) and sensing-related parameters (e.g., network sensing coverage, probability of missed detection of an event, sensor failure probability, *etc*.).

Despite the fact that some protocols or mechanisms required by operational needs (items (ii)–(vi)) are already reported in scientific literature, their validation in real-world WSNs applications is still pending. Despite all these difficulties, the use of directional antennas in wireless sensor networks has already proved several advantages: it improves the transmission reliability, increases the spatial reuse, extends the transmission range or decreases the overall network power consumption. Moreover, directional antennas offer sensor nodes additional control over signal strength and interference, which allows the use of optimization techniques for providing higher network throughput and transmission reliability. Last but not least, the directional antennas provide significant advantages in coping with various security threats.

Studying the factors that can boost the effectiveness of such devices when coping with security attacks, we found that narrowing the radiation region of antennas favors both of the abovementioned types of approaches (direct and indirect). By this the probability of eavesdroppers/jammers to be outside the radiation zone is increased and, moreover, the localization based on signal’s angle of arrival becomes more accurate. The endeavor to narrow the radiation region for directional antennas is not a simple task knowing that the antenna’s size increases with the increase of angular resolution [17]. From the information security point of view, employing directional antennas for communication purposes inside WSN opens up a wide spectrum of new research opportunities as follows:

(a) *Involving directional antennas in coping with other malicious attack type.* While the configuration of their radiation pattern can inherently mitigate the effects associated to eavesdropping or jamming, the directional antennas can be involved in identifying, mitigating or even eliminating the security risks associated to other malicious attacks using angular information (signal’s direction of arrival). For this, the key word is “localization”, so any malicious attack that can be addressed using localization-based techniques (*i.e.*, position verification) can be a valid target for future research. Relevant examples in this context are the selective forwarding attack [68] or the Hello flood attack [69].
(b) *Using directional antenna-based localization mechanisms to detect security attacks on other localization schemes.* The WSN’s localization infrastructure is susceptible to an assortment of malicious attacks [70] that can endanger the network’s proper functioning. Effective localization schemes based on the use of GPS devices or lateration-based algorithms can be automatically validated using angulation-based approaches relying on intrinsic angular information provided by directional antennas.
(c) *Eliminating the consequences of several attacks by benefiting from the longer transmission range of directional antennas.* A concrete example can be the case of sensor nodes or groups of sensor nodes isolated from the rest of the network due to various malicious attacks (e.g., jamming, node capturing attack, resource depletion attack, *etc*.). In this kind of situation the nodes can find alternative paths to regain the connectivity to the rest of WSN by contacting nodes that are further away.
(d) *Using sensor nodes with both directional and omnidirectional antennas to solve complex security issues inside WSN.* Such an approach could combine the potential advantages brought by the two antenna types. In this case, strategies to switch from one type of antenna to the other have to be design in order to maximize the WSN capability to timely discover and eliminate the security risks.
(e) *Coordinating the mechanisms based on the use of directional antennas with other security related technique.* Coping with the increased diversity of security threats that affect wireless sensor networks, demands the use of a complex ensemble of methodologies and protocols. The integration of security mechanisms based on directional antennas in an overall security system it’s not a simple task due to a series of factors including the power, communication and computational constraints, the heterogeneity of sensor nodes, the unattended or hostile nature of the WSN environment, *etc.*
(f) *Extending the research field by addressing the security problems of more complex versions of WSNs,* where the sensor nodes are endorsed with mobility (e.g., mobile wireless sensor networks [71] or airborne wireless sensor networks [72]) or where the sensor nodes coexist with other wireless node types (wireless sensor and actuator networks [73] or even wireless sensor, actuator and robot networks [74]).
(g) *Fusing information received from directional antennas and from other devices (e.g., sensors) for coping with security threats.* In many cases, the network nodes are able to obtain supplementary information that can be used to mitigate the security attack risks. Routing information, list of neighboring nodes together, locations and battery energy levels of neighboring nodes or successive sensor measurements are only few examples of information that can be utilized in this context to mitigate the security risks. For example, multimedia sensor nodes equipped with video and audio capture capabilities can fuse such information with the ones obtained from directional antennas to address security-related issues.

## 6. Conclusions

The use of directional antennas for equipping WSN nodes arises from the need to optimize energy consumption, to raise the quality of transmissions or to decrease the number of hops due to longer transmission ranges. Besides this, directional antennas can be seen as a valuable resource for reducing the security risks that inherently affect WSNs’ operation. In this paper, after surveying the prototypes of directional antenna suitable for WSN nodes, we presented the state of the art in mitigating the security risks associated to eavesdropping, jamming, Sybil and wormhole attacks. Even though research in this area is still in a beginning stage, the results are encouraging, demonstrating the need for further theoretical and experimental investigation. Certainly, future studies should include new research topics including the need to cope with other types of malicious attacks, to consider the potential benefits of using both directional and omnidirectional antennas on the same sensor nodes, to combine the strategies based on the use of directional antennas with other security-related methods, or to expand the research area to other more complex varieties of WSNs.

## Figures and Tables

**Table 1:** Omnidirectional *vs.* directional antenna comparison.

Characteristic	Omnidirectional Antenna	Directional Antenna
Energetic efficiency	Lower	Higher
Broadcasting direction	All	Desired
Transmission range	Lower	Higher
Node orientation	Not required	Required
Price	Lower	Higher
Dimensions	Smaller	Bigger
Transmission security	Lower	Higher
Collisions	More	Less

**Table 2:** Directional antenna prototypes for WSNs.

Research	Frequency	Antenna’s Structure	Mote Platform
Leang and Kalis [30]	868 MHz	Two horizontal or vertical wire antennas and a reflective SPDT switch	SensorDVB
Nilsson [31,32]	2.4 GHz	Electronically switched parasitic element antenna	TMote Sky
Giorgetti *et al.* [33]	2.4 GHz	A box-like structure of four coaxially fed planar patch antennas	TelosB
Liang *et al.* [34]	2.4 GHz	Active cylindrical frequency selective surface	VirtualSense
Catarinucci *et al.* [12]	2.4 GHz	Radiation structure made of eight microstrip antennas using rectangular two-element patch antenna arrays and a vertical half-wavelength dipole antenna	STM32W-EXT
Catarinucci *et al.* [35,36]	2.4 GHz	Four identical antennas, containing two quarter-wavelength l-shaped slot antenna elements, disposed in a symmetrical planar structure	STM32W-EXT
Felemban *et al.* [37]	2.4 GHz	6-Sectored antennas having an overlap of 120 degrees in azimuth	Nano-Qplus

**Table 3:** Summary of research on the use of directional antennas in WSN security.

Research	Attack	Directional Antenna Involvement	Short Description
Dai *et al.* [41,42]	eavesdropping	direct	Establishes eavesdropping models for omnidirectional and directional antennas, proving that directional antennas perform better
Li *et al.* [43]	eavesdropping	direct	Analysis the effects of using directional antennas upon eavesdropping probability from the attacker’s perspective
Kim *et al.* [44]	eavesdropping	direct	Employs special nodes (defensive jammers) equipped with directional antennas in mitigating the eavesdropping attacks
Noubir [45]	jamming	direct	Proves the efficiency of directional antennas in jamming circumstances by comparing the network connectivity index
Panyim *et al.* [46]	jamming	direct	Proposes a combined strategy that uses pre-distributed cryptographic keys in conjunction with sensor nodes able to switch from omnidirectional to directional antennas anytime a jamming attack is detected
Staniec and Debita [47]	jamming	direct	Suggests two simultaneous defense strategies: equipping the nodes with directional antennas and establishing a superior limit of the duty cycle
Newsome *et al.* [48]	Sybil attack	indirect	Provides a list of possible defenses against Sybil attacks, underlining the efficiency of position verification tactics
Suen and A. Yasinsac [49]	Sybil attack	indirect	Uses nodes equipped with GPS and directional antennas to locate the Sybil nodes
Bhatia *et al.* [50]	evil-twin attack	indirect	Employs nodes equipped with four-sector directional antennas to detect malicious nodes using Hyperbolic Position Bounding algorithm
Vaman and Shakhakarmi [51]	Sybil attack	indirect	Proposes an integrated key-based Strict Friendliness Verification of neighboring nodes
Rabieh *et al.* [52]	Sybil attack	indirect	Identifies Sybil attacks using directional information, public key cryptography and hash function applied to trial messages
Hu and Evans [53]	wormhole attack	indirect	Proposes three approaches to mitigate wormhole attacks, the basic idea being to maintain an accurate list of trusted neighbors
Shi *et al.* [54]	wormhole attack	indirect	Proposes a Secure Neighbor Discovery scheme for wireless networks with a centralized network controller; the approach uses signature based authentication, transmission time information and directional information
Vaman and Shakhakarmi [51]	wormhole attack	indirect	Proposes a mechanism based on symmetric node ids, round trip response times and real time location information obtained by directional antennas
